# Morphological Changes Induced by Bipolar Radiofrequency Ablation in Thyroid Nodules – a Preclinical *Ex Vivo* Investigation

**DOI:** 10.17925/EE.2016.12.02.85

**Published:** 2016-08-28

**Authors:** Daniel Igor Branovan, Mikhail Fridman, Maxim Lushchyk, Valentina Drozd, Olga Krasko, Olga Nedzvedz, Nikolay Shiglik, Larisa Danilova

**Affiliations:** 1. Project Chernobyl, New York, United States; 2. Minsk City Clinical Oncologic Dispensary, Minsk, Belarus; 3. Belarusian Medical Academy of Post-Graduate Education, Minsk, Belarus; 4. United Institute of Informatics Problems, Minsk, Belarus; 5. Belarusian State Medical University, Minsk, Belarus

**Keywords:** Radiofrequency ablation, thyroid gland, nodular goitre

## Abstract

**Introduction:** Recently, radiofrequency ablation (RFA) has been increasingly used for the treatment of thyroid nodules. However, immediate morphological changes associated with bipolar devices are poorly shown. **Aims:** To present the results of analysis of gross and microscopic alterations in human thyroid tissue induced by RFA delivered through the application of the original patented device. **Materials and methods:** In total, there were 37 surgically removed thyroid glands in females aged 32–67 at presentation: 16 nodules were follicular adenoma (labelled as ‘parenchymal’ solid benign nodules) and adenomatous colloid goitre was represented by 21 cases. The thyroid gland was routinely processed and the nodules were sliced into two parts – one was a subject for histological routine processing according to the principles that universally apply in surgical pathology, the other one was used for the RFA procedure. **Results:** No significant difference in size reduction between parenchymal and colloid nodules was revealed (p>0.1, t-test) straight after the treatment. In addition, RFA equally effectively induced necrosis in follicular adenoma and adenomatous colloid goitre (p>0.1, analysis of variance test). As expected, tumour size correlated with size reduction (the smaller the size of the nodule, the greater percentage of the nodule volume that was ablated): r=-0.48 (p<0.0001). **Conclusion:** The results make it possible to move from *ex vivo* experiments to clinical practice.

The latest epidemiological studies have demonstrated that the prevalence of thyroid nodules in adults has reached an alarming 50–67%.^1,2^ Nonsurgical, minimally invasive modalities, such as ethanol ablation, laser ablation, radiofrequency ablation (RFA), and high-intensity focused ultrasound have also been reported to be effective options in treating thyroid nodules.^3^ Since the first reported series in 2006, there have been numerous studies showing efficacy and safety in treating benign ‘cold’ and ‘hot’ thyroid nodules.^4,5^ In addition, minimally invasive tumour treatment with radiofrequency induced thermotherapy has been proposed for the management of recurrent nodal metastases in patients with well-differentiated thyroid carcinoma.^6–8^

Contrary to the growing experience in using non-surgical procedures in thyroid nodule treatment, immediate morphological changes produced by ablation are rarely investigated.^9^ Therefore, this study aimed to present the results of analysis of acute gross and microscopic alterations in human thyroid tissue induced by RFA.^10^

## Material and methods

The Ethical committee of Minsk City Clinical Oncologic Dispensary approved the study design. All patients included in the research were diagnosed with benign solid thyroid nodules according to the diagnostic protocol (physical, laboratory, ultrasonography evaluation, fine-needle aspiration cytology). In total, there were 37 surgically removed thyroid glands in females aged 19–73 at presentation: 16 nodules were follicular adenoma (labeled as ‘parenchymal’ solid benign nodules) and adenomatous colloid goitre was represented by 21 cases. The thyroid gland was routinely processed and the nodules were sliced into two parts – one was a subject for histological routine processing according to the principles that universally apply in surgical pathology, the other one was used for the RFA procedure. The maximum time span between the thyroid surgery and experiments was 15 minutes. All RFA applications were performed at room temperature.

**Figure 1:**
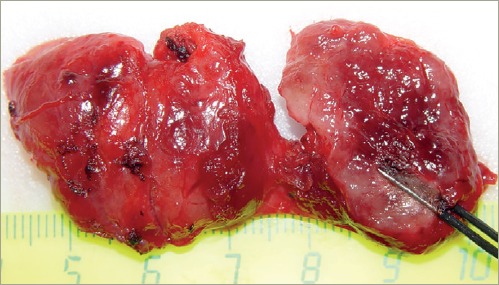
A bipolar radio frequency ablation device inserted into the thyroid gland Necrotic areas are clearly visible along the active tip of the device.

During the RFA procedure, thermal energy was delivered through a bipolar applicator^10^ with a diameter of 1.3 mm (18-gauge), a shaft length of 102 mm, and an active tip length of 10 mm (see *Figure 1*).

RFA ablation was performed with an exposure time of 20 seconds. Power of 20 watts was applied. Higher power rates were omitted in the preliminary tests because the resultant lesions insignificantly differed from those that were gained after applying power of 20 watts (due to the effect of carbonisation). Lower power rates are unsuitable due to longer exposure times.

**Figure 2:**
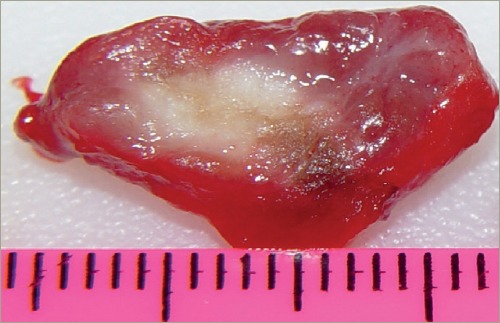
The grey-brown coagulated tissue is dry and thick

Once RFA was been finished, the nodules were cut open along the electrode axis. The lesions were macroscopically inspected along axial and transversal planes. Clearly demarcated portions of the visibly damaged area were regarded as necrosis (see *Figure 2*).

Each axial and transversal diameter was measured. The primary nodule bed dimension was calculated from the measurements, assuming a radially symmetrical lesion shape and employing the formula for an ellipsoid.

## Statistical analysis

We estimated the size of the surgically removed nodule as a surrogate endpoint of √(a×b), where a and b are bi-dimensional diameters, and labelled it ‘primary nodule size’, which is calculated in millimetres. In the same way we estimated the size of the necrotic foci, which were labelled as ‘lesion size’.

Nodule size reduction was expressed in percentage and defined as a ratio of the ‘lesion size’ to primary nodule size. Correlation was performed by the Spearman test. We compared differences in size of necrosis and nodule size reduction in groups of tumours using a one-way analysis of variance (ANOVA) test.

A p-value <0.05 was considered statistically significant. Analyses were conducted using R version 3.1.3 software (R Project for Statistical Computing, www.r-project.org).

## Results

All nodules (n=37) were divided by their size: up to 1 cm in diameter versus more than 1 cm and morphological characteristics (see *Table 1*). No significant difference in size reduction between parenchymal and colloid nodules was revealed (p=0.2571, t-test). Besides, RFA equally effectively induced necrosis in parenchymal and colloid nodules (p=0.2337, ANOVA-test). As expected, tumour size correlated with size reduction (the smaller size of the nodule, the greater percentage of nodule was ablated): r=-0.48 (p<0.0001).

**Figure 3:**
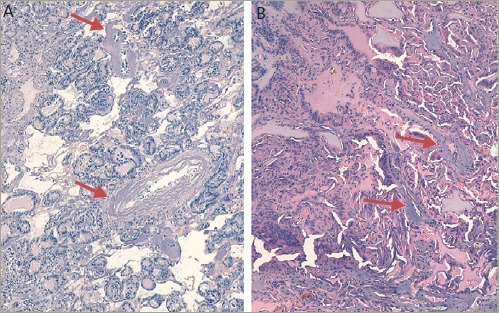
Thyroid nodule architectonic after radiofrequency ablation application A: stroma in follicular adenoma showed extensive edematous/loose changes; B: goitre tissue was totally disorganised, many follicle walls were ruptured, clearly visible eosinophilic vitreous-like colloid was located inside macro-follicles and in stroma as well. Indicated by arrows (A and B): lumens of blood vessels inside the nodules are narrowed, vascular walls considerably thickened owing to extravasated plasma protein and deposition of basement membrane material, smooth muscle cells totally disappeared. Slides stained with haematoxylin and eosin, x40.

**Figure 4:**
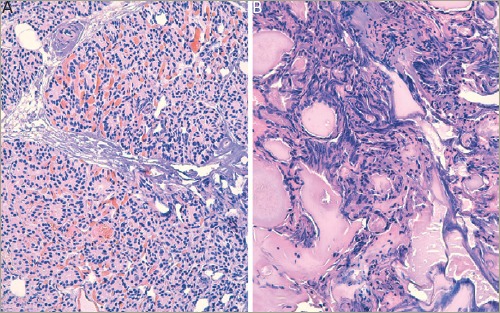
Severe stromal degeneration and accentuated lobulation of the thyroid nodule accompanied by significant reduction in capillary blood flow and tissue plethora (A); the chief cells had nuclear shrinkage and increased basophilia as in follicular adenoma (A) and in goitre (B) as well Slides stained with haematoxylin and eosin, x100.

**Figure 5:**
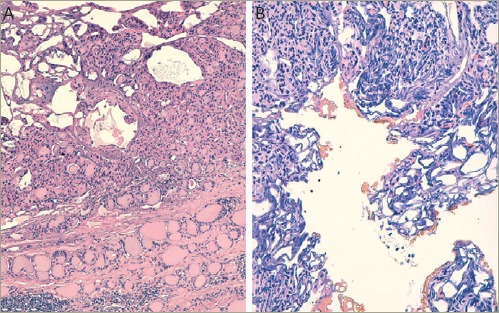
Cavities were formed due to ablation (A); in more pronounced lesions small cavities merged into larger bubbles (B) Slides stained with haematoxylin and eosin, A: x40, B: x100.

**Figure 6:**
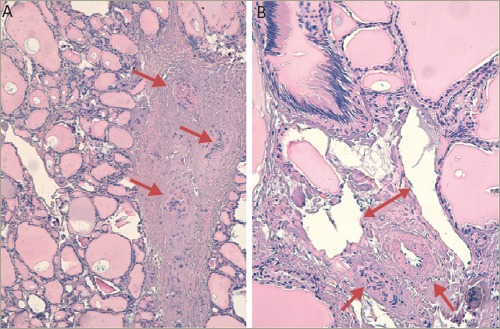
Vascular reactions on the periphery of the nodule: small veins were markedly narrowed (arrows), (A and B); in contrast, lymphatics were dilated (double arrow) (B) Slides stained with haematoxylin and eosin, A: x40, B: x100.

Pathological examination revealed destructive changes (see *Figures 3–6*). The degree of these was graded as mild, moderate or severe. The thermal artifacts extended and altered the shape and size both of parenchymal structures such as glands, cells and nuclei, and vessels. A thin layer of thermal coagulation at the margin of nodules characterised mild artifacts. Moderate artifacts consisted of a layer of more noticeable thermal coagulation, visible in the demarcation area. Severe artifacts were marked by the presence of: extensive necrotic areas, wrinkling of the tissue, swelling and blurring of cell details. Also, depending on the calibre of the vessels, some of them appeared spastic (venules), and others were reciprocally dilated (small lymphatics).

**Table 1:** The results of radiofrequency ablation according to nodule size and morphological characteristics

Morphological characteristics	Nodule size	
	≤10 mm	>11 mm
Number of nodules		
Parenchymal solid benign lesions	11	5
Colloid hyperplastic benign lesions	9	12
Primary nodule axial size (A), mm, mean (SD)		
Parenchymal solid benign lesions	7.4 (1.8)	14.4 (3.4)
Colloid/hyperplastic benign lesions	7.4 (1.8)	13.6 (2.5)
Axial size of necrotic focus, mm, mean (SD)		
Parenchymal solid benign lesions	5.9 (1.7)	9.6 (2.7)
Colloid /hyperplastic benign lesions	6.2 (2.0)	8.6 (2.2)
Size reduction, %, mean (SD)		
Parenchymal solid benign lesions	78.6 (18.4)	68.1 (17.5)
Colloid/hyperplastic benign lesions	84.9 (21.2)	64.5 (15.5)

## Discussion

Owing to the use of new ultrasound technologies in clinical practice, it appears that thyroid nodular disease is a widespread illness. Although most newly diagnosed nodules are benign, others are the subject of long-term observation, keeping in mind the risk of malignancy.^11,12^ The published results of a prospective cohort analysis showed that the prevalence of thyroid nodular disease increases with advancing age, whereas the risk of malignancy in a newly identified nodule declines.^13^

There is still much debate about the risks of malignancy in thyroid nodules in the Belarusian population exposed to radiation due to the Chernobyl catastrophe.^14^ A high prevalence of thyroid nodules combined with growing numbers of additional risk factors for thyroid malignancy and uncertainty of molecular testing are resulting in increasing surgical activity for benign thyroid nodules. Considering that there can be complications following thyroid surgery, the introduction of minimally invasive approaches for treatment of this widespread nodular disease is vital.^15^ In addition, long-existing, slow-growing benign nodules raise a question – whether to choose to ‘wait and observe’, or take a more active approach.^16^

There are sufficient data in the literature on the clinical efficacy of RFA – thermo-lesion is well controlled, and performed effectively.^17–19^ As for the comparison of monopolar or bipolar RFA – no conclusive data are available yet.

Our results reveal that straight after the treatment with bipolar RFA reproducible lesions of sufficient clinical size (nodular size reduction from 56.1% to 84.9%) are formed in a shorter time (from 10–15 minutes with the monopolar technique to 20–30 seconds with the bipolar RFA technique).

Therefore, the bipolar RFA method could be applicable for outpatient practice in the management of benign thyroid nodules of clinically significant diameters, decreasing the risks of the possible future malignancy of long-existing and continuously observed lesions. We demonstrated the effectiveness of RFA on gross and microscopic levels. Morphological features were more significant in thyroid lesions sized up to 1 cm; multiple RFA procedures are required to ablate larger nodules. On the other hand, further *in vivo*, long-term observation studies with possible comparative analyses of monopolar versus bipolar RFA procedures are desirable.
